# Associations between dietary habits and bipolar disorder: a diet-wide mendelian randomization study

**DOI:** 10.3389/fpsyt.2024.1388316

**Published:** 2024-05-10

**Authors:** Junyao Li, Renqin Hu, Huirong Luo, Yanwei Guo, Zheng Zhang, Qinghua Luo, Pingyou Xia

**Affiliations:** ^1^ Department of Psychiatry, The First Affiliated Hospital of Chongqing Medical University, Chongqing, China; ^2^ Yongchuan District Mental Health Center, Chongqing, China

**Keywords:** diet, bipolar disorder, Mendelian randomization, nutrition, prevention

## Abstract

**Background:**

Diet/nutrition is critically important in the pathogenesis, progression, and treatment outcomes of various mental disorders. Current research predominantly focuses on the role of diet in the development and treatment of depression, with less attention given to the relationship between diet and Bipolar Disorder (BD).

**Method:**

We employed Mendelian Randomization (MR) to investigate the relationship between 28 dietary habits and BD. An analysis was conducted using publicly available genome-wide association study data from the UK Biobank dataset. Various dietary habits were analyzed as exposures with BD as the outcome, mainly using the Inverse Variance Weighted (IVW) method.

**Results:**

Intake of non-oily fish and sponge pudding both have a positive association with BD. Oily fish, dried fruit, apples, salt, and cooked vegetables intake also appeared potentially risky for BD, although the possibility of false positives cannot be ruled out. Sensitivity analysis further confirmed the robustness of these findings.

**Conclusion:**

Our research provides evidence of a relationship between various dietary habits and BD. It underscores the need for careful dietary management and balance to reduce the risk of BD, suggesting caution with dietary preferences for fish and sponge pudding. Furthermore, more detailed studies are needed to further understand the potential impacts of high-sugar and high-protein diets on BD development.

## Background

Bipolar Disorder (BD) is a mental disorder marked by alternating episodes of mania or hypomania and depression. It affects approximately 1%-4% of the population (1). The correlation between diet and various physical diseases is well-established and documented, and in recent years, this has extended to the exploration within psychiatric disciplines (2, 3). In managing BD, nutrition plays a critical role due to two key reasons. Firstly, individuals with BD often exhibit poor health habits that lead to appetite and energy fluctuations, core characteristics of the disorder, typically linked to suboptimal nutritional choices (4). Secondly, BD is closely linked with several physical health concerns, including obesity, metabolic syndrome, cardiovascular and respiratory diseases, and endocrine disorders. Notably, cardiovascular diseases are responsible for nearly 40% of deaths among BD patients (5). These health issues are attributed to the disease’s inherent pathophysiological mechanisms, such as systemic factors like brain and bodily immune inflammation, glucose-insulin imbalances, oxidative stress imbalance, mitochondrial dysfunction, premature or accelerated aging, as well as the side effects of medications and unhealthy lifestyles (6–11). Diet, nutritional patterns and specific nutrients may have an impact on these mechanisms, such as excessive sugar intake causing oxidative stress and inflammation, and omega-3 fatty acids have anti-inflammatory activity and affect neuroplasticity (12, 13).

For individuals with BD, while pharmacological and psychological treatments are accessible, they often fall short of fully alleviating symptoms. In this context, nutrition has emerged as a promising area for intervention. As existing evidence emphasizes, the brain necessitates specific nutrients, including energy (a crucial component of the total energy in food), lipids, vitamins, macro and micronutrients, antioxidants, and catalysts for neurotrophins synthesis, to function and maintain structural integrity (14). A comprehensive meta-analysis, grounded in this theoretical framework, has reviewed the current evidence on nutrition and BD, and found that the effects of supplementing with creatine, carnitine, vitamin D, inositol, or N-acetylcysteine on BD are variable (15). Additionally, studies related to probiotics and Coenzyme Q10 have been indicated to yield positive outcomes. Therefore, dietary patterns and related nutritional practices indirectly influence the development and progression of BD, positioning them as pivotal targets for therapeutic intervention and prevention.

The majority of existing observational studies are influenced by confounding factors, leaving the connection between diet and BD somewhat ambiguous. This gap indicates that the significance of nutrition in the disease course of BD may not be fully recognized and utilized. Future research needs to delve deeper into the specific links between dietary habits and BD to clarify the pathophysiological mechanism of BD onset and the efficacy and mechanisms of dietary interventions in BD treatment. Such research is crucial for developing more comprehensive BD prevention and treatment strategies, and providing more targeted dietary recommendations for patients.

Psychiatric disorders are subject to potential reverse causation between risk factors and the disease, complicated by numerous confounding factors (e.g., genetic and environmental risks) that limit our understanding of their pathophysiology. This complexity makes it particularly difficult to identify the risk factors and mechanisms of mental illness, leading to inconsistent diagnoses, such as with bipolar disorder (BD), which is often misdiagnosed because it predominantly presents with depressive symptoms (16). Each person is naturally assigned a genetic variant that may affect risk factors in different ways, which makes Mendelian Randomization (MR) particularly valuable in the study of psychiatry (17). At present, more studies on the application of MR focus on Major Depressive Disorder (MDD) and Schizophrenia, and less focus on BD (17). In nutritional epidemiological studies, it is very challenging to minimize confounding biases that can lead to inconsistent relationships between diet and BD. Recently, several Genome-Wide Association Studies (GWAS) studies have shown that dietary habits are heritable traits (18, 19). Thus, the MR Method of applying genetic variation to instrumental variables can provide valuable exploration in this field. MR serves as a valuable tool for discerning the causal effects of various exposures on outcomes, utilizing genetic variations as instrumental variables. This approach effectively mitigates potential confounders and reverse causation issues, thereby complementing observational studies and randomized controlled trials. Therefore, MR may be a suitable study design to assess the impact of diet on disease or health outcomes (20). Existing evidence suggests a potential causal link between daily dietary habits and BD, particularly with the consumption of wheat, avoiding eggs, sugar, dairy products, and the maintenance of high plasma caffeine levels (21, 22). However, these studies only explored a few dietary habits as exposures in relation to BD, neglecting a comprehensive investigation. Additionally, when selecting usable SNPs, it did not exclude those that were associated with confounding traits, potentially causing a bias on results. Although some studies have summarized related dietary information, there is still a lack of systematic research and rigorous design examining dietary habits in the context of BD.

This study utilizes MR to conduct a thorough investigation into the relationship between 28 prevalent dietary habits and BD. Exploration examines various dietary patterns to ascertain which may act as protective factors or pose risks for BD. The goal of this approach is to guide general population towards making healthier dietary choices and to avoid inappropriate nutritional intake to increase BD risk, while also providing recommendations about the quality of food sources. Moreover, this study holds the potential to enhance causal interpretations within observational epidemiology, thereby contributing new insights to clinical therapeutic interventions and prevention strategies, ensuring a more seamless and rigorous exploration.

## Method

**Figure 1:**
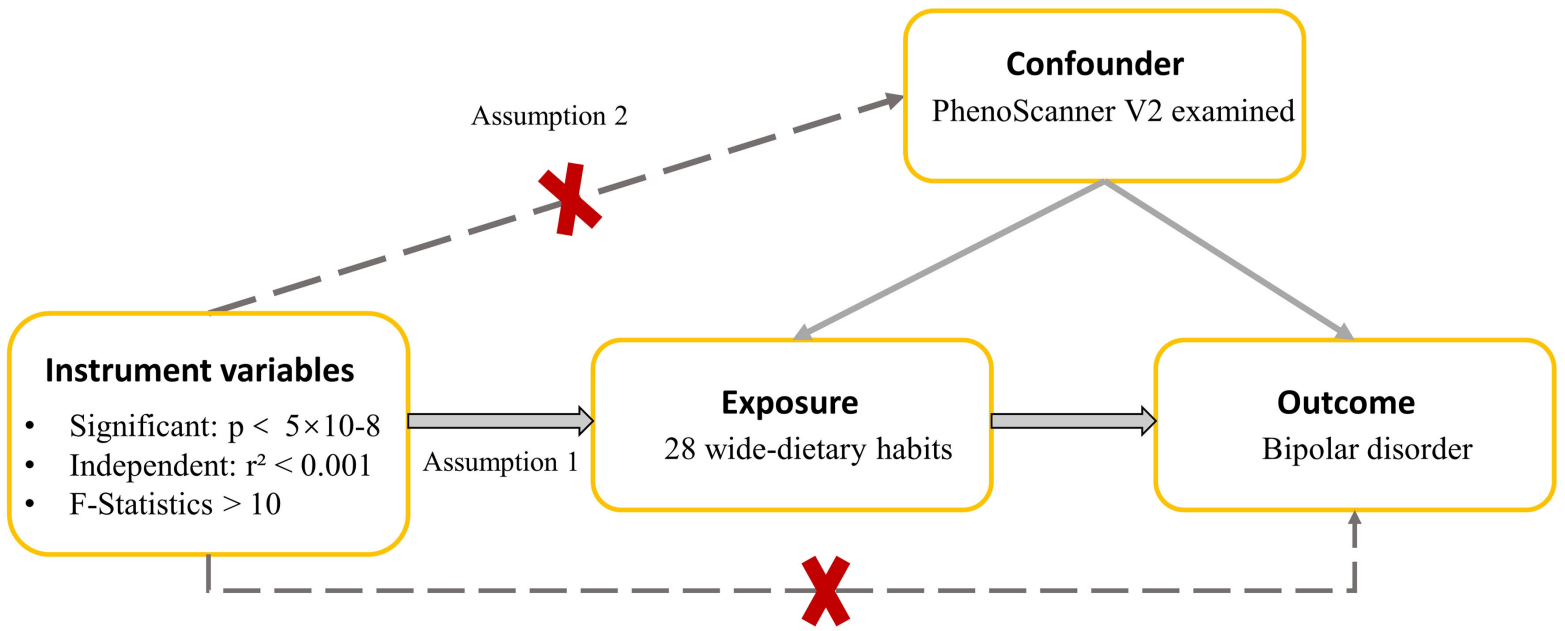
A directed acyclic graph is employed to depict the hypothesized influence of dietary habits on BD. The use of a dotted line in this graph signifies the possibility of a direct causal relationship or a pleiotropic effect existing between the exposure and the outcome.

**Figure 2:**
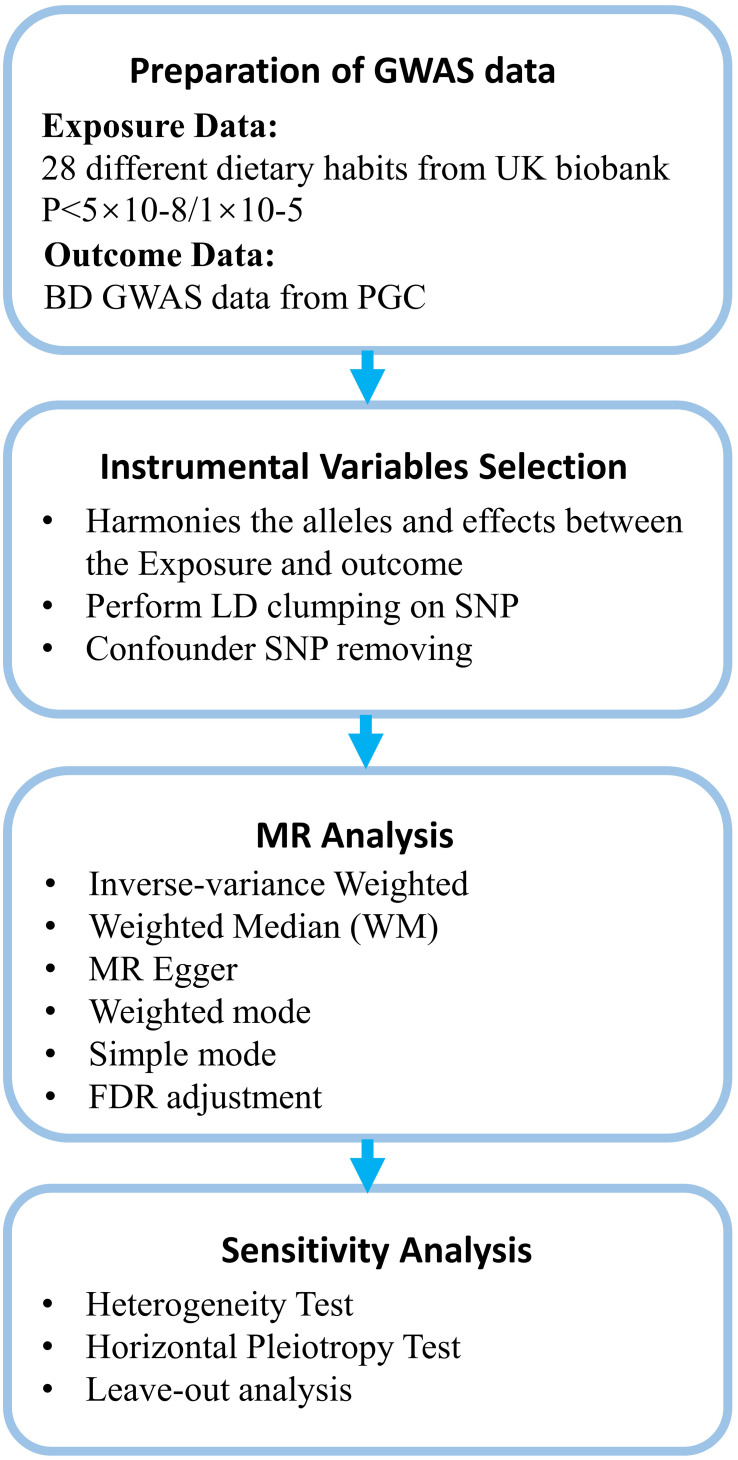
Summary of the experimental protocol.

This study investigates the relationship between dietary habits and BD. **Figure 1** provides an overview of the research design and data sources. **Figure 2** offers a summary of the experimental protocol used in our study. We exclusively utilize publicly available GWAS summary statistics, eliminating the need for additional ethical approvals or informed consent.

### Exposure data

In this study, we have specifically focused on 28 distinct dietary habits as the exposure variables, carefully selected based on prior research (11, 14). Exposures covering eight categories, included processed meat intake, poultry intake, beef intake, pork intake, lamb mutton intake, non-oily fish intake, oily fish intake, bread intake, dark chocolate intake, cake intake, cheese intake, sponge pudding intake, cereal intake, white rice intake, fresh fruit intake, dried fruit intake, apple intake, banana intake, berry intake, cooked vegetable intake, salad raw vegetable intake, tea intake, coffee intake, alcohol intake frequency, fizzy drink intake, milk intake, pure fruit/vegetable juice intake, and salt added to food. These data were derived from the GWAS conducted by the UK Biobank, a large-scale prospective study involving approximately 500,000 participants aged between 38 and 73, providing genetic and phenotypic information (23). Comprehensive descriptions of the research design, participants, and quality control methods have already been published (24). The data for each dietary pattern we considered include integer variables (i.e., the average number of cups of coffee consumed per day) and categorical variables (i.e., the frequency of poultry consumption). Unreasonable responses were excluded at the time of data submission. The summary data for GWAS dietary habits were collected from the MRC-IEU’s OpenGWAS project, organized by Elsworth et al., and maintained by the MRC-IEU. Detailed information is listed in **Table 1**.

**Table 1:** Detailed information on the GWAS datasets used in this MR study.

Exposure	Food types	Trait			Sample Size	Consortium
ID						
ukb-b-6324 ukb-b-8006 ukb-b-2862 ukb-b-5640 ukb-b-14179	Meat	Processed meat intake Poultry intake Beef intake Pork intake Lamb mutton intake			461,981 461,900 461,053 460,162 460,006	MRC-IEU (Elsworth et al.)
ukb-b-17627 ukb-b-2209	Seafood	Non oily fish intake Oily fish intake			460,880 460,443	
ukb-b-11348 ukb-b-16139 ukb-b-3433 ukb-b-1489 ukb-b-12067	Dairy and Sweet	Bread intake Dark chocolate intake Cake intake Cheese intake Sponge pudding intake			452,236 64,945 64,949 451,486 64,949	
ukb-b-15926 ukb-b-14690	Grain	Cereal intake White rice intake			441,640 64,949	
ukb-b-3881 ukb-b-16576 ukb-b-4070 ukb-b-5362 ukb-b-8887	Fruit	Fresh fruit intake Dried fruit intake Apple intake Banana intake Berry intake			446,462 421,764 64,949 64,949 64,949	
ukb-b-8089 ukb-b-1996	Vegetable	Cooked vegetable intake Salad raw vegetable intake			448,651 435,435	
ukb-b-6066 ukb-b-5237 ukb-b-5779 ukb-b-2832 ukb-b-2966 ukb-b-337	Beverage	Tea intake Coffee intake Alcohol intake frequency Fizzy drink intake Milk Pure fruit/vegetable juice intake			447,485 428,860 462,346 64,949 64,943 64,949	
ukb-b-8121	Food additive	Salt added to food			462,630	

Outcome						
ID	Trait		Sample Size	Consortium		
ieu-b-5110	Bipolar Disorder bip2021		413,466	PGC Bipolar Disorder Working Group of PGC		

PGC, the Psychiatric Genomics Consortium. Population consisted of individuals of European descent.

### Outcome data

We used the GWAS summary data on BD outcomes from the Psychiatric Genomics Consortium. The BD-related SNPs were obtained from a large GWAS meta-analysis of 57 cohorts from Europe, North America, and Australia, including a total of 41,917 BD cases and 371,549 controls of European ancestry (25). Cases of BD were identified using international consensus criteria [Diagnostic and Statistical Manual of Mental Disorders (DSM-IV), International Statistical Classification of Diseases 9th Revision (ICD-9) or International Statistical Classification of Diseases 10th Revision (ICD-10)], established using structured diagnostic interviews, clinician-administered checklists or medical record review. Most control groups were screened for psychiatric disorders using ICD codes. Overall, 64 genomic loci associated with BD were identified through this analysis.

We carefully examined the sample source. Exposures were derived from the UK Biobank dataset, whereas outcomes were obtained from pooled BD GWAS data across 57 cohorts in total. Within BD data, the UK Biobank dataset accounted for 1454/41917 of the total BD sample, suggesting that overlap with the population of exposures is less likely to cause bias risk. Meanwhile, we also used pleiotropy test to help verify the sample overlap bias risk.

### Selection of instrumental variables and statistical analysis

In this study, instrumental variables (IVs) for dietary exposures were sourced from the UK Biobank. The selection criteria for relevant SNPs included: (1) Initial screening of SNPs robustly associated with 28 dietary habits at genome-wide significance (P<5×10^-08). (2) Clumping of identified SNPs for linkage disequilibrium (LD) using PLINK with a stringent cutoff of R^2^ = 0.001 within a 10,000 kb window, referencing European samples from the 1000 Genome Project (26, 27). In cases of LD effects among SNPs, the SNP with the lowest P value was retained. (3) To address potential weak instrument bias, F-statistics were calculated following established methodology, excluding SNPs with an F-statistic below 10 (28, 29). Post harmonization of exposure and outcome datasets, with palindromic and weak instrumental variants removed, the remaining SNPs were utilized for MR analysis. If fewer than 3 exposure-related SNPs were available, the selection threshold P value was adjusted to 1×10^-5. Additionally, PhenoScanner V2 was employed to assess associations between selected SNPs and potential confounders (P < 5×10^-05) (30). SNPs associated with confounders were excluded in further analyses.

To ensure the validity of an MR study, an IV must adhere to three crucial assumptions: (1) the relevance assumption, indicating that the IVs is associated with the exposure; (2) the independence assumption, indicating that the IVs is not influenced by any confounding factors; and (3) the exclusion restriction assumption, indicating that the IVs affects the outcome exclusively through its impact on the exposure (31). In this study, the first assumption was scrutinized by calculating the proportion of explained variance (R²) and the F statistics of IVs (Calculation formula and relevant results are shown **Supplementary Table S1**) (31–35). F>10 was considered to be a strong IV (33). We used the PhenoScanner V2 to remove from the selected SNPs those that were associated (P<5×10–05) with confounders (REPRESENTING traits that are different from exposure) (List of Confounders are shown in **Supplementary Table S2**). In short, we evaluate and ensure the adequacy of IVs by calculating the F statistics and determining the R² after removing the specific SNPs obtained by confounding factors. The third assumptions can be partially met if horizontal pleiotropy (an IV influences the outcome through another exposure other than the one under investigation) is absent (31). Horizontal pleiotropy could be tested by whether the MR-Egger intercept is significantly different from 0 (31). P>0.05 for the MR-Egger intercept (p value of pleiotropy) provides no evidence that horizontal pleiotropy is present (31).

We utilized Inverse Variance Weighted [IVW, random-effects (RE)], MR Egger, Weighted Median (WM), and Weighted Mode to analysis. The primary method for analysis was IVW, which uses meta-analysis techniques to combine the Wald ratios of individual SNPs, and assuming that the IVs affect the outcome solely through a specific exposure. Overall, this approach is predicated on the assumption of no average pleiotropic effects and is regarded as highly efficient for such analyses (36). Heterogeneity in MR analysis refers to inconsistencies in the estimates derived from different IVs, reflecting the compatibility of the instrumental variables with the causal inference being made. We used Cochran’s Q test, with random-effects IVW employed for analysis when p < 0.05 was considered indicative of high heterogeneity.

Our study involved a total of 28 exposures in 8 dietary categories, which was moderate in size for most MR studies. Considering the widespread nature of dietary habits in everyday life, it required a more cautious and scientific study design. For this reason, we applied an FDR (False Discovery Rate) correction to the P-values to balance exploration and conservatism of study. Subsequently, to ensure the accuracy of our findings, we also implemented a more rigorous Bonferroni correction. We considered the differences to be statistically significant when P<0.05. High-confidence findings were those that survived multiple-testing adjustment. To validate the reliability of the estimates, we performed a leave-one-out sensitivity analysis by recalculating the overall effect size and removing each SNP one at a time until reaching significant results for the primary outcome. All analyses were conducted using the TwoSampleMR package (version 0.5.9) in R (version 4.3.2).

## Results

**Table 1** provides comprehensive information about each participating GWAS study. We assessed the impact of various dietary exposures on the outcome, and, in all cases, the F statistics of the identified SNPs surpassed the empirical threshold of 10, ranging from 21.30 to 48.27. This finding suggests that the obtained results are less susceptible to deviations caused by weak IVs. Detailed information of MR results and sensitivity analysis can be found in **Supplementary Table S3**.

### Effect of dietary habits on BD

**Figure 3:**
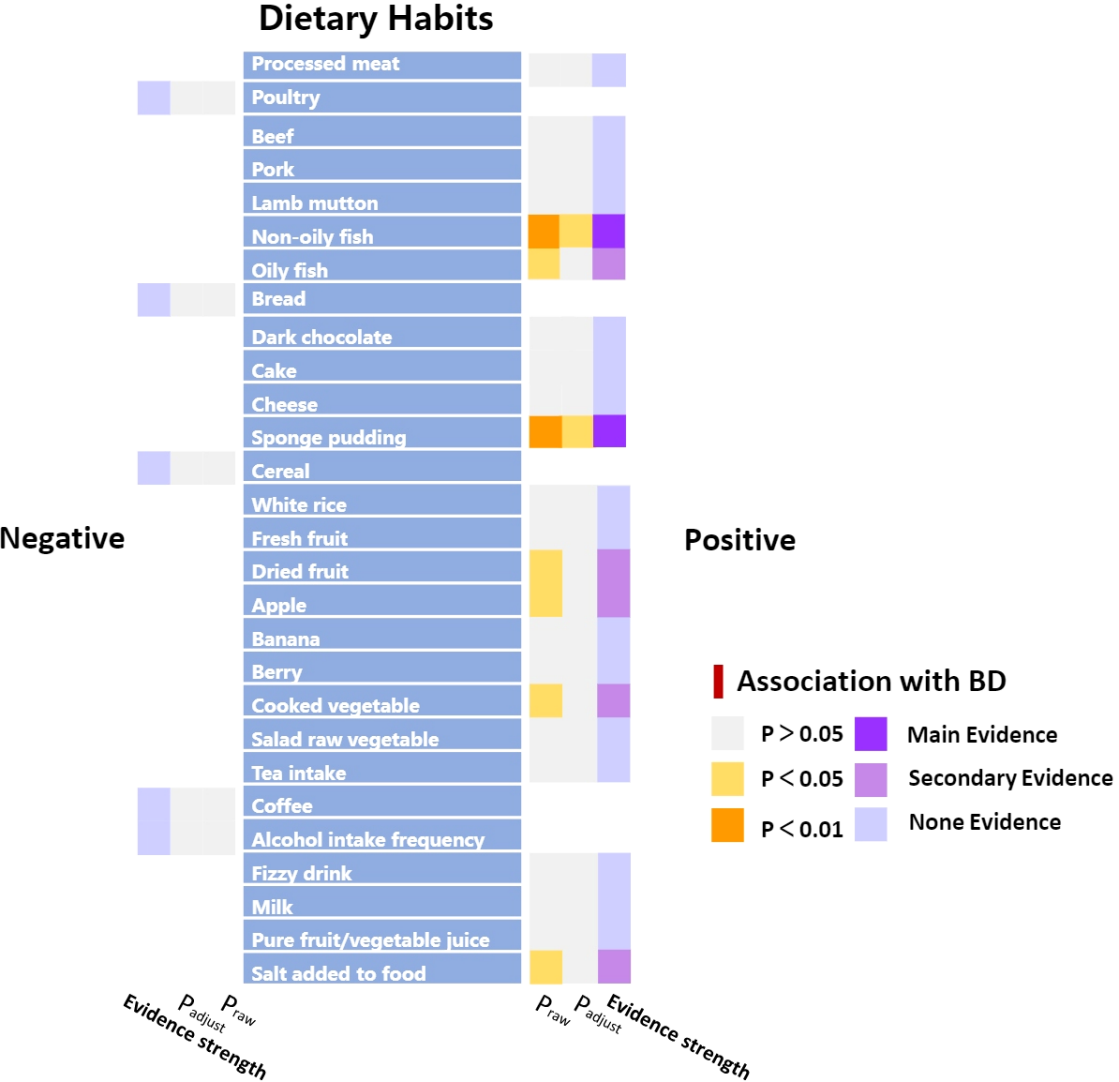
Summary results of association between dietary habits and BD according to the IVW method. “Positive” represents the factors that have a risky effect on BD, and “Negative” represents the factors that have a protective effect on BD, as defined by the beta value in the MR analysis.

**Figure 4:**
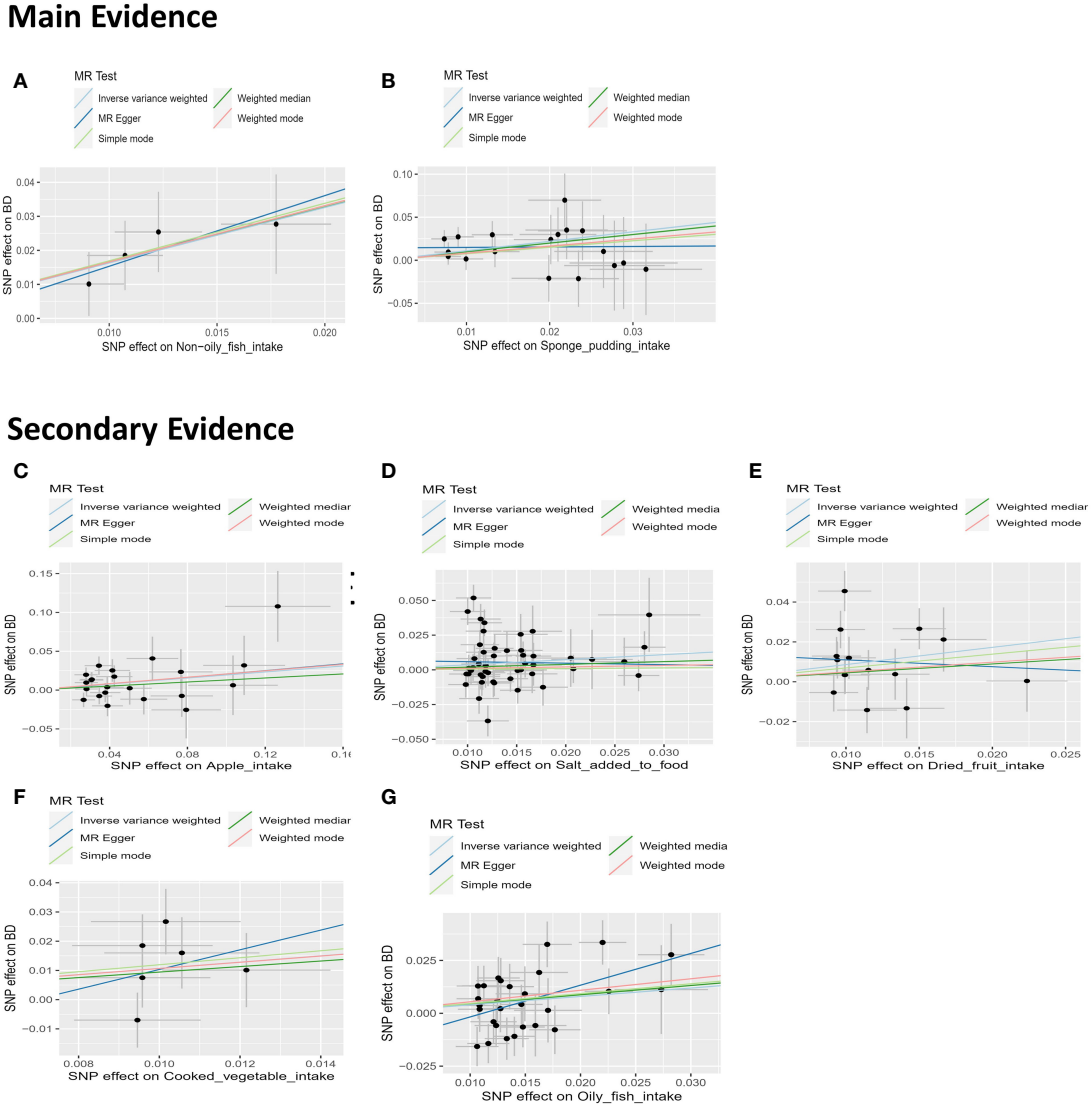
Scatter plots depicting the results of MR analyses investigating the association between dietary habits and BD. Each line in the plot represents a different MR method, and the slope of each line represents the estimated association between the two variables. **(A)** Scatter plot between non-oily fish intake and BD; **(B)** Scatter plot between Sponge pudding intake and BD; **(C)** Scatter plot between Apple intake and BD; **(D)** Scatter plot between salt added in food and BD. **(E)** Scatter plot between Dried fruit intake and BD; **(F)** Scatter plot between cooked vegetable intake and BD; **(G)** Scatter plot between Oily fish intake and BD.

The impact of dietary habits on BD is summarized in **Figure 3**. The relationships between 28 dietary habits and BD were determined based on the p-values obtained from the IVW method (**Table 2**). Our findings indicate that BD was found to be correlated with seven dietary habits, including intake of apples (β=0.194, SE=0.087, P_raw_=0.026), salt (β=0.364, SE=0.175, P_raw_=0.037), cooked vegetable (β=1.070, SE=0.488, P_raw_=0.028), non-oily fish (β=1.629, SE=0.466, P_raw_=0.0004), oily fish (β=0.398, SE=0.166, P_raw_=0.016), dried fruits (β=0.863, SE=0.355, P_raw_=0.015), and sponge pudding (β=1.100, SE=0.336, P_raw_=0.001). Among these, two remained significant even after FDR, reflecting that BD has a strong association and actual effect with the intake of non-oily fish (β=1.629, SE=0.466, P_adjust_=0.010) and sponge pudding (β=1.100, SE=0.336, P_adjust_=0.011). We conducted a more rigorous Bonferroni follow-up, and the results also supported the findings of FDR. The results from the WM method largely align with those from the IVW method. Scatter plots were also constructed to visualize the main results (**Figure 4**). In MR analysis, significant results from analyzing a single exposure and outcome suggested a causal link can be reported. However, when analyzing additional exposures, the significance may diminish after adjusting for multiple comparisons, hinting at a potential false-positive. This suggests the exposure doesn’t hold up under stricter statistical scrutiny, indicating the initially observed association’s strength has weakened. Evidence levels were categorized based on these findings (see **Figure 3**), with primary evidence showing both original and adjusted P-values as significant, secondary evidence showing only the original P-value as significant, and no evidence indicating no significant association. This structured approach allows for a cautious interpretation of results.

**Table 2:** MR results of the IVW method for the association of dietary habits with BD.

Exposure	Number of SNPs	β	SE	p-value	Adjusted p-value (FDR)	Bonferroni (0.05/28)
Processed meat	9	0.017	0.264	0.947	0.770	–
Poultry	62	-0.096	0.204	0.636	0.692	–
Beef	3	0.541	0.593	0.361	0.561	–
Pork	5	0.132	0.975	0.891	0.759	–
Lamb mutton	14	0.150	0.548	0.783	0.735	–
Non-oily fish	4	1.629	0.466	0.0004**	0.010*	**√**
Oily fish	28	0.398	0.166	0.016*	0.084	–
Bread	12	-0.297	0.271	0.273	0.491	–
Dark chocolate	17	3.972	15.32	0.795	0.737	–
Cake	16	0.110	0.148	0.456	0.617	–
Cheese	24	0.101	0.254	0.690	0.709	–
Sponge pudding	18	1.100	0.336	0.001**	0.011*	**√**
Cereal	11	-0.110	0.434	0.799	0.738	–
White rice	16	12.796	9.420	0.174	0.381	–
Fresh fruit	18	0.257	0.398	0.518	0.647	–
Dried fruit	15	0.863	0.355	0.015*	0.081	–
Apple	22	0.194	0.087	0.026*	0.100	–
Banana	14	0.007	0.121	0.950	0.770	–
Berry	17	0.237	0.125	0.058	0.171	–
Cooked vegetable	6	1.070	0.488	0.028*	0.103	–
Salad raw vegetable	6	0.341	1.111	0.758	0.728	–
Tea	15	0.145	0.199	0.465	0.622	–
Coffee	13	-0.035	0.311	0.909	0.762	–
Alcohol intake frequency	36	-0.022	0.128	0.860	0.752	–
Fizzy drink	17	0.144	0.140	0.301	0.515	–
Milk	30	0.075	0.155	0.628	0.689	–
Pure fruit/vegetable juice	15	0.227	0.136	0.096	0.254	–
Salt added to food	48	0.364	0.175	0.037*	0.117	–

*p<0.05 **p<0.01 √p<0.05/28 –p>0.05/28.

Significant p-values have been highlighted in bold.

In our study, evidence of pleiotropy was found in the intake of white rice, suggesting that this evidence is somewhat ambiguous and merely indicative. While no evidence of pleiotropy was found for the other exposures, it was verified that the genetic variation used as instrumental variables in MR Studies could not affect the results through other pathways. This also helped to some extent to verify the low risk of bias caused by overlapping samples. Our results demonstrated a widespread heterogeneity effect, but using IVW as the main analytical method helps to control for the effects of heterogeneity by emphasizing the contribution of more precise studies and assuming a straightforward relationship between genetic instruments and the traits studied. In this way, IVW method yielding a more stable and reliable estimate of the causal effect, even when heterogeneity among studies exists.

**Figure 5:**
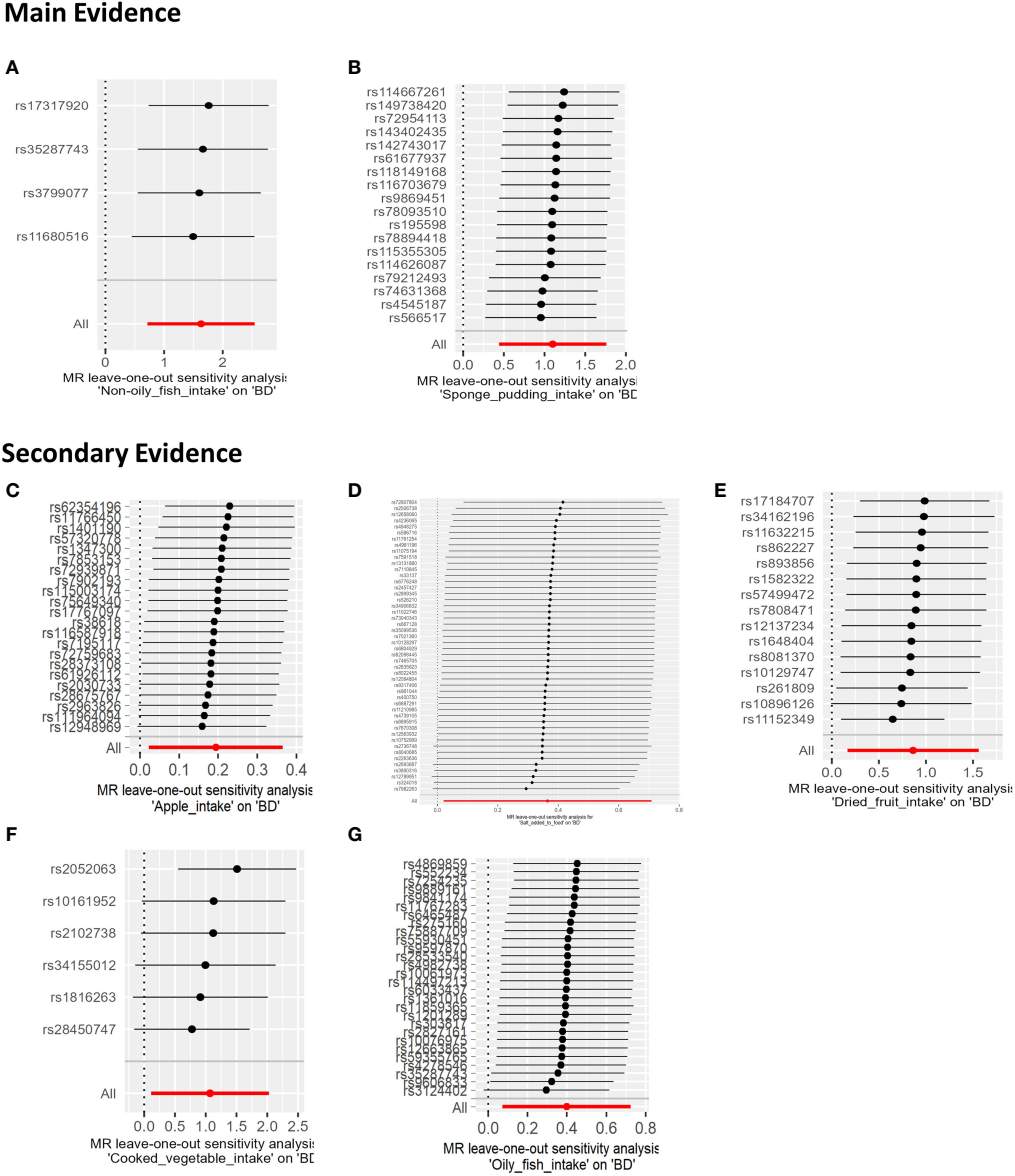
The results of a Leave-one-out analysis on MR. Each black line in the figure corresponds to the outcome of the MR analysis when one SNP is removed from the analysis, while the remaining SNPs are used on the left. **(A)** Leave-one-out analysis between non-oily fish intake and BD; **(B)** Leave-one-out analysis between Sponge pudding intake and BD. **(C)** Leave-one-out analysis between Apple intake and BD; **(D)** Leave-one-out analysis between salt added in food and BD.; **(E)** Leave-one-out analysis between Dried fruit intake and BD; **(F)** Leave-one-out analysis between cooked vegetable intake and BD; **(G)** Leave-one-out analysis between Oily fish intake and BD.

**Figure 6:**
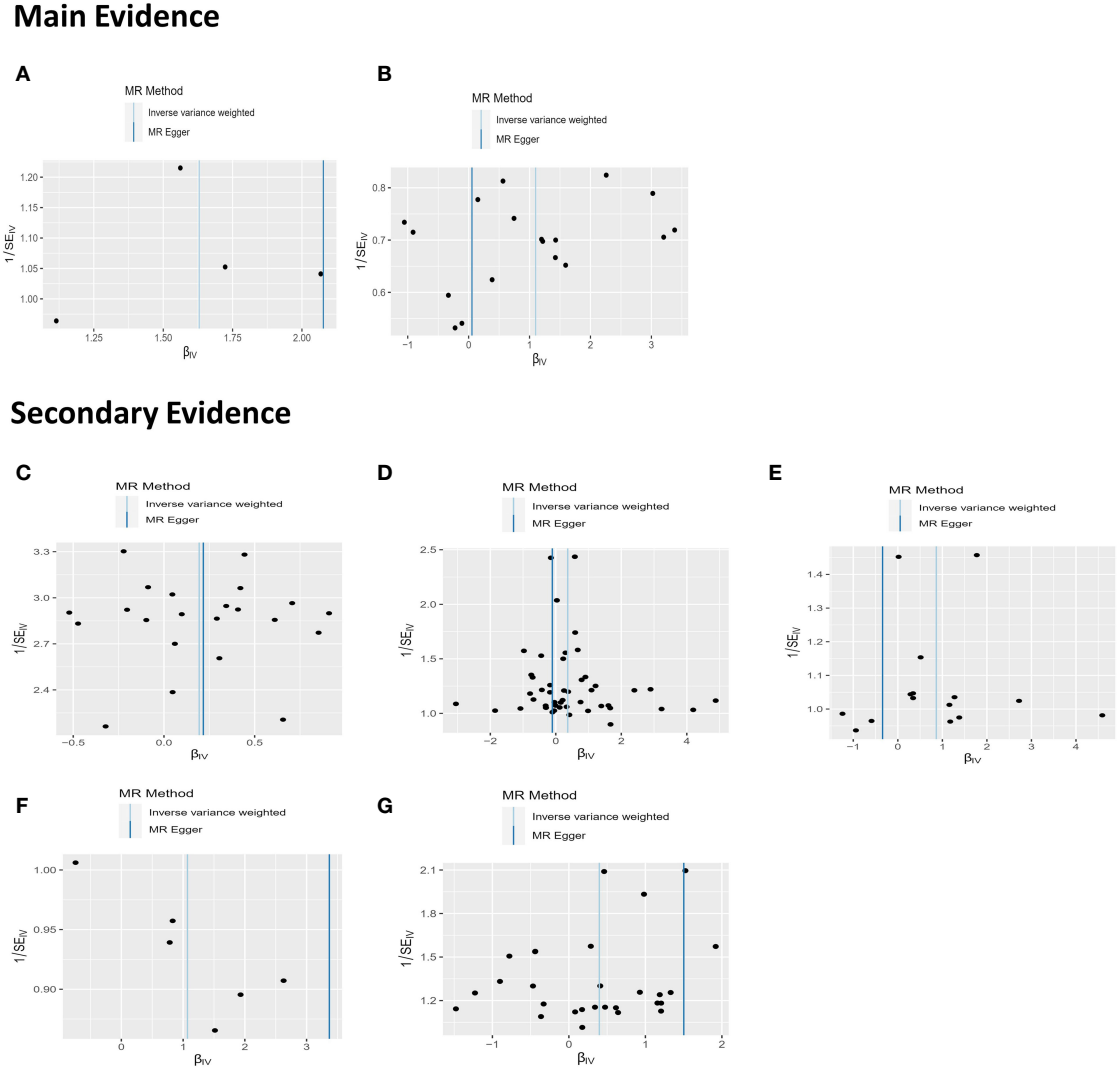
The results of funnel plots on MR. **(A)** Funnel plot between non-oily fish intake and BD; **(B)** Funnel plot between Sponge pudding intake and BD; **(C)** Funnel plot between Apple intake and BD **(D)** Funnel plot between salt added in food and BD. **(E)** Funnel plot between Dried fruit intake and BD; **(F)** Funnel plot between cooked vegetable intake and BD; **(G)** Funnel plot between Oily fish intake and BD.

In our sensitivity analysis of the main results, we conducted a leave-one-out analysis, excluding one significant SNP at a time. For all significant associations with BD, the omitted estimates also had statistical significance (**Figure 5**). Funnel plots further confirmed the credibility of the results obtained using the IVW method (**Figure 6**).

## Discussion

While the pathophysiology of BD remains elusive, research over the past decade has shown that BD is associated with disorders in several areas, including alterations in various brain structural regions, neuroendocrine and monoaminergic transmission, immune/inflammatory processes, mitochondrial activity, oxidative stress, and neural progression (37, 38). In addition to the significant role of genetics, dietary habits in daily life are also considered important factors affecting the onset of BD, for example nutritional deficiencies in Omega-3 polyunsaturated fatty acids (PUFAs) and iron are thought to be related to its onset, development and exacerbation (13, 39). The effect of diet on neuroendocrine hormones and neurotransmitters may explain the BD happen and appetite changes in individuals with mood disorders, but this area still requires further research for a deeper understanding and validation. With proper dietary guidance, may influencing the course of BD in positive way and may reduce the risk of diet-related diseases. For instance, patients with BD who also have Type II diabetes or glucose intolerance face a higher risk of adverse (chronic) BD course compared to those without blood sugar control issues (40). Moreover, changes in weight (both loss and gain) are associated with the incidence of manic and depressive episodes (11). In this study, we conducted an analysis of the UK Biobank dataset to investigate the impact of 28 dietary habits on BD.

Interestingly, our study showed that the intake of non-oily fish is positively associated with BD. Fish is widely considered to be beneficial for health, being rich in fatty acids (such as Omega-3 and Omega-6), proteins, vitamins (such as D and B complex), and minerals (such as iron, calcium, and zinc). Current epidemiological data have already shown a negative correlation between the incidence of BD and fish consumption, highlighting the importance of Omega-3 fatty acids abundant in fish and involved in regulating brain signals and reducing oxidative stress (13, 41–43). Although most studies support the role of fish intake in the prevention and intervention of BD, the issue remains controversial. An English study found that the consumption of fish did not improve mood in general population (44). A clinical research indicates that restricting animal-based foods like meat, fish, and poultry can improve short-term mood symptoms (45). Another study suggests that fish oil supplements are healthier than fish and that purified supplements may provide the benefits of omega-3 fatty acids (46). Furthermore, non-oily fish intake may even increase the risk of the MDD, as reported by a previous MR study (47). Nevertheless, the authors did not provide insights into the mechanisms behind this finding, instead focusing only on discussing the reasons for the differences in the relationships between BD and the intake of oily versus non-oily fish. Overall, while previous studies haven’t provided conclusive evidence, most indicate a beneficial impact. Our findings still seem to challenge the widely held view that fish intake is beneficial for BD. One possible explanation for this risk factor is that a high-protein diet may lead to stress responses of hormones, neurotransmitters and toxic metabolites, thus participating in the pathogenesis of BD (48). Key components of proteins, amino acids—especially tryptophan, phenylalanine, and tyrosine—are crucial in mental health for their role in synthesizing neurotransmitters like dopamine and serotonin, which are essential for brain cell communication (49). Changes in serotonergic neurotransmitters have been shown to be involved in the pathogenesis of BD (50, 51). Tryptophan is the main precursor of serotonin, and its concentration determines the rate of serotonin synthesis in the brain (52–54). High protein intake could hinder the ability of amino acids related to neurotransmitters to cross the blood-brain barrier due to competition for carriers with tryptophan, and serotonin production may be reduced (55). Another potential mechanism for the onset of BD is the impact of homocysteine (Hcy, a high level of Hcy is generally considered toxic to cells), which is highly positively correlated with a diet rich in animal protein (56–58). The interaction of Hcy with glutamate transmission is considered to be a major mechanism related to BD. Hcy and its oxidative metabolites, homocysteic acid, act as agonists within the N-methyl-D-aspartate (NMDA) receptor (59). Long-term activation of the NMDA receptor leads to an increase in calcium ion inflow, producing neurotoxic effects (60). The increased release of glutamate by presynaptic neurons enhances the NMDA receptor current in postsynaptic neurons, potentially leading to excitotoxicity (61). This is consistent with findings of elevated glutamate levels in BD, where excessive glutamate is associated with increased mitochondrial reactive oxygen species and excitotoxicity. Additionally, high levels of Hcy may have toxic effects on dopaminergic neurons, and dysfunction of dopaminergic neurons is closely related to BD (62). Finally, when Hcy acts as a methyl donor for S-adenosylmethionine during activation, it suggests that abnormal DNA methylation may also be involved in the pathogenesis of BD (63). In our study, we discovered that the intake of non-oily fish had a more pronounced impact on BD than oily fish. This difference may be due to the lower levels of Omega-3 fatty acids in non-oily fish, which are known for their protective effects (13). Consequently, the reduced content of these fatty acids in non-oily fish might make it more susceptible to negative effects. These potential mechanisms may mediate the relationship between fish intake and BD risk. Other studies, including a Japanese study linking plant protein with reduced depression symptoms and observational research linking high animal protein intake in women with a higher risk of mental illnesses, support these findings (64, 65). A meta-analysis that included eight observational studies showed that meat consumption may be associated with a slightly higher risk of depression (66). While there is no direct evidence linking animal protein to an increased risk of BD, the importance of considering protein sources and overall dietary balance in general population management need to be explored in future.

In our study, we did not find a relationship between BD and meat (diets rich in animal protein). Here, we propose several possible explanations: 1) Antagonistic and synergistic interactions among various nutrients (67). For instance, meat, which is rich in fats and animal proteins, may impact nutritional efficiency and the body’s utilization of these nutrients due to interactions during digestion, absorption, and metabolism. Factors such as digestion speed (the presence of fat can delay gastric emptying, thereby slowing protein digestion and absorption), metabolic effects (combined intake may affect the body’s metabolic state), and energy density (high energy from fats may alter the demand for proteins as an energy source) illustrate that nutrient interactions are complex and require further exploration to understand the underlying mechanisms that support the different associations between meat and fish consumption and BD risk. 2) Genetic and metabolic differences. Individuals prone to BD may respond more strongly to fish protein due to genetic backgrounds or metabolic differences. These explanations also seem to apply to other similar situations.

Our study also demonstrated a positive link between sponge pudding intake and BD. Sponge pudding is a dessert made from a mixture of flour, eggs, sugar and milk, which provides a large amount of nutrients such as sugar and saturated fat. High-sugar, high-saturated fat dietary preference is often observed in the patients with BD, supporting a correlation with symptoms (68, 69).This kind of diet may be associated with disruptions in monoaminergic activity. Animal studies have indicated that the combined intake of fat and sugar reduces dopamine D2 receptor signaling (70). High-sugar diets affect catechol-O-methyltransferase activity, while high-fat diets impact the expression of glutamate decarboxylase and brain GABA concentrations (71, 72). A human study found that high-calorie, high-fat, high-sugar snacks lowered serotonin transporter levels in the hypothalamic region (73). The intake of a high-fat diet may also increase the likelihood of immune-inflammatory events (i.e., western dietary pattern can lead to elevated levels of C-reactive protein and interleukins), neuroprogression (i.e., high-fat diet reduces BDNF expression and decreases the number of newly generated cells in the hippocampal dentate gyrus), and mitochondrial damage (i.e., rats on a high-fat diet show significantly reduced antioxidant defense capabilities) (74–76). Previous studies have also established a connection between sugar intake and BD, associating high sugar consumption with an increased risk of BD and impulsive behaviors (12, 22). A nature reward from sugar is another evolutionary adaptation, as it drives humans to seek out and consume sugar from the supply, similar to how the consumption of ripe fruit and honey can increase survival chances in times of food scarcity due to its important role in storing fat (77, 78). These individuals with the greatest fat stores may have a stronger evolutionary advantage when it comes to survival. Thus, sugar cravings likely imparted a strong evolutionary advantage. However, humans have never adapted to the intense reward after consuming highly refined added sugars. Excessive sugar intake may contribute to the development and progression of BD by inducing neuroadaptive changes in the brain’s reward system, effects of metabolic byproducts, oxidative stress, obesity, and inflammation (12). Long-term studies have indicated that sugar intake can stimulate our brain’s reward system, activate dopamine-related regions (posterior midbrain, dorsolateral/orbitofrontal cortex), and lead to an imbalance of neurotransmitters (79). In BD patients, regional specificity of dopaminergic activity is different compared to general population (80). Pharmacological evidence also supports the role of dopamine in BD, which is commonly increased by using anti-manic drugs and antidepressants (80, 81). We have found that apples may also be a potential risk factor for BD. Another possible mechanism explanation is that the intake of fructose (richly found in apple) leads to elevated serum uric acid (a metabolite of fructose), which could associate with onset of BD (82). Clinical studies have indicated that serum uric acid levels vary significantly between different phases of BD. Patients in manic phases often exhibit higher levels of serum uric acid, whereas those in depressive phases typically show lower levels (83). Further treatment studies have also substantiated uric acid’s role in precipitating manic episodes (84). Additionally, studies on psychosocial behavior have linked high uric acid levels with traits like impulsivity, sensation-seeking, hyperactivity, and other personality temperaments that are commonly observed in individuals with BD (85). Uric acid can increase the stress response stimulation of the hippocampus, a region in the human brain that initiates stress responses by activating the pituitary-adrenal axis, ultimately leading to the secretion of cortisol (86). This hormone is responsible for upregulating many other inflammatory markers, including cytokines in the body (87–89). Overall, the increase in uric acid due to fructose intake, thereby, enhances hippocampal stress response, leading to brain inflammatory responses and foraging behaviors. This could partially explain the potential association between apple intake and the risk of BD, as well as the preference among BD patients for high-sugar diets. Unlike apple, dried fruit not only contains naturally occurring fructose but also often includes highly refined added sugars to enhance its flavor. The strong sweet reward system impact is even greater than cocaine (77). This unnatural reward has outpaced our self-control mechanisms, not only exacerbating mood swings but also contributing to addiction (90, 91). There is a growing body of evidence suggesting that diets rich in artificial added sugar of processed food are harmful to mental health, linked to increased severity of depressive symptoms in those with mood disorders and life stress in people experiencing psychosis (92, 93).

The association between salt intake and BD remains not fully explored. Recently, a meta-analysis suggested that salt intake was higher in people with BD compared to controls (94). Possible mechanism to explain is that a high-salt diet can lead to decreased cerebral blood flow, elevated stress hormone levels (such as cortisol), cardiovascular health damage, and potentially result in cognitive decline, heightened psychological stress, emotional issues related to organic diseases, poor sleep quality, and the emergence of anxious and depressive moods (95, 96). Additionally, our study presents a positive link between the consumption of cooked vegetables and BD, although existing research does not directly address the impact of cooked meals on BD. Different cooking methods, like frying, which typically involve high-calorie and high-fat intake, may trigger inflammatory responses or oxidative damage, recognized as risk factors for BD (15, 97). It is worth noting that we did not find evidence for frequent consumption of coffee, tea and alcohol in BD patients as often pointed out in observational studies, which may be more mediated by social environment and physiological factors mediating the relationship between disease and risk factors (98–100).

To our knowledge, this is the first study to report the potential risk effect of a broad range of dietary habits on BD. Our research suggests that two dietary habits (intake of non-oily fish and sponge pudding) are positively associated with BD. Given the presence of false positives, secondary evidence including the intake of salt, oil-fish, cooked dishes, apples, and dried fruits may be positively associated with BD. Previous systematic reviews have explored the relationship between diet and BD, suggesting and encouraging the adoption of a healthy dietary lifestyle, including daily intake of fruits, cooked vegetables, seafood, and whole grains (15). However, many factors may confound these relationships, including socioeconomic status, physical health, and lifestyle habits such as exercise and smoking, along with potential residual bias, insufficient power, and lack of temporality in data analysis. Large-sample MR studies have somewhat corrected these issues by using genetic variations to simulate randomization, thereby reducing the influence of confounding factors and producing more reliable results. Surprisingly, our conclusions contradict previous beliefs about the positive and beneficial effects of dietary habits. Based on current evidence, it may be too speculative to conclude that avoiding high-protein and high-sugar diets can prevent BD. Moreover, given the potential for pleiotropy bias in MR analysis, and the linear relationship between SNPs, exposures, and outcomes assumed in MR analysis, our results should be interpreted with caution. The relationship between diet and mental health is complex, involving multiple factors and mechanisms. Before making specific dietary recommendations, further observational studies are needed to explore the relationship and consider the interactions among nutrients. Identifying dietary habits that may protect against BD is critical for prevention.

## Limitation

Despite this study being one of the most comprehensive MR studies to assess the role of diet on BD, our study involves several limitations. First, its methodology, mainly focusing on individuals of European descent, may limit its generalizability. Secondly, while we deliberately focused on examining the relationships between specific dietary exposures and outcomes based on certain literature, our approach to selecting diets was not entirely systematic and may lack comprehensiveness. Therefore, future research should embrace a broader spectrum of dietary explorations to enhance our understanding. Thirdly, the original data utilized in the MR analysis was not segregated by gender, lacked standardized diagnostic criteria, and did not account for comorbidities, which could introduce gender-specific biases and variabilities into the analysis. To refine the accuracy of future studies, it is advised that GWAS datasets should include gender-specific data and apply standardized diagnostic and inclusion criteria with greater precision and caution. Finally, the complexity and interplay of dietary components suggest that focusing solely on individual nutrients or food groups may be flawed. Nutrient interactions and antagonisms of varying degrees within various diets make the relationship between dietary habits and mental health even more complex. Before making specific dietary recommendations, further research is needed to explore the specific relationships between nutrients involved in exposures, as well as the underlying biological mechanisms.

## Conclusion

This study identified correlation between specific dietary habits and BD. High intake of sponge pudding and non-oily fish positively associated with BD. Other dietary habits, such as consumption of apples, dried fruits, cooked vegetables, salt, and oily fish, appeared potentially risky for BD, although the possibility of false positives cannot be ruled out. In summary, our research provides evidence of relationships between various dietary habits and BD. In daily life, it is necessary to be cautious about high fish intake and sponge pudding intake. Moreover, it will be crucial to investigate the impact of diets high in sugar (including various types of sugars) and protein on the pathogenesis of BD and their influence on the disease’s onset. Understanding their roles could significantly contribute to strategies for primary prevention of BD.

However, it is important to note that the relationship between diet and mental health is complex, involving multiple factors and mechanisms. Based on the current evidence, it may be overly speculative to suggest that avoiding high-protein or high-sugar diets could prevent BD. Therefore, our findings should be applied with caution, and more observational studies are needed for further exploration.

## Data availability statement

The original contributions presented in the study are included in the article/**Supplementary Material**. Further inquiries can be directed to the corresponding authors.

## Author contributions

JL: Conceptualization, Data curation, Investigation, Methodology, Validation, Writing – original draft, Writing – review & editing. RH: Investigation, Methodology, Validation, Writing – review & editing. HL: Investigation, Methodology, Validation, Writing – review & editing. YG: Conceptualization, Investigation, Writing – review & editing. ZZ: Writing – review & editing.. QL: Conceptualization, Project administration, Supervision, Writing – review & editing. PX: Conceptualization, Project administration, Supervision, Writing – review & editing.
